# Myasthenia gravis masquerading as acute stroke: a case report

**DOI:** 10.11604/pamj.2020.37.305.27032

**Published:** 2020-12-02

**Authors:** Sarath Menon Ramachandra Menon, Namith Ranjith Mokkath

**Affiliations:** 1 Department of Neurology, Sree Narayana Institute of Medical Sciences (SNIMS), Kunnukara, India

**Keywords:** Myasthenia gravis, acute stroke, reversible tongue atrophy, acetyl choline receptor antibody, case report

## Abstract

Among non-iatrogenic neuromuscular disorders, myasthenia gravis remains the most prevalent. Diagnosing this disorder may become challenging in certain cases such as in patients with coexisting comorbid illnesses and non-specific clinical symptoms. This is a case of atypical myasthenia gravis in a middle-aged hypertensive male, who initially presented symptoms suggestive of an acute ischemic stroke. Upon later investigation, prompted by persistent symptoms, the patient was found to have AchR antibodies and had the rare finding of a fissured and atrophied tongue (reversible on treatment). It is a well-known fact that brainstem strokes can present with bulbar weakness resulting in aspiration pneumonitis, as was with the clinical presentation in the below mentioned report. Due to the initial misdiagnosis, he had received medical therapy aimed towards stroke management and prevention. Further investigation leading to a definitive diagnosis, was followed by medical therapy with neostigmine, pyridostigmine and oral prednisolone, leading to significant improvement in symptoms. Hence as a mandatory measure, while dealing with a case of a new onset of weakness, especially in cranial musculature, myasthenia gravis must not be excluded from the list of differential diagnosis. Myasthenia gravis (MG) is a potential “stroke mimic” especially in the elderly. However, due to recent change in trends of stroke statistics, this disease should be considered a possibility even in younger patients.

## Introduction

We report a case of atypical myasthenia gravis in a hypertensive male, who presented with semiology, suggestive of a young stroke. Diagnosing this disease may become challenging in certain cases: in patients with atypical clinical symptoms and those with comorbidities attributing to a specific etiology. The symptoms on presentation were acute onset of dysphagia, nasal regurgitation on eating and dysarthria. He was initially diagnosed with and treated for an acute stroke with bulbar weakness with associated aspiration pneumonitis. Continued evaluation was carried out due to persistence of symptoms even after discharge from hospital, finally clinching the correct diagnosis of MG. This case carries special significance since it was misdiagnosed as young stroke, followed by treatment with anticoagulants and blood thinners. It is to be noted that such measures and other aggressive therapies such as intravenous thrombolysis might lead to serious adverse outcomes in cases where they are not indicated. It is also worth noting that this patient was found to have a fissured/furrowed and atrophied tongue (a rare finding in AchR antibody positive MG).

## Patient and observation

A 48-year-old male who presented to the neurology out-patient department (OPD) in October 2019 and the cascade of events from the time of diagnosis and management till follow up had gained our attention. The patient's complaints were, dysphagia, nasal regurgitation on eating and dysarthria for two days. He had no significant medical history expect for systemic hypertension which was not being regularly followed up or monitored. On examination, he had intact higher mental functions, normal orientation, and comprehension and was fully alert and conscious with a Glasgow Coma Scale (GCS) of 15/15. Cranial nerve examination revealed palatal weakness with an impaired gag and cough reflex; a nasal quality in speech too was observed along with normal extra-ocular movements. Further neurological examination was consistent with normal motor, sensory and cerebellar functions. While auscultating the patient's chest, normal heart sounds were heard and scattered crepitations were appreciated over bilateral lung fields.

Being a hypertensive male, as this patient had an acute presentation of symptoms and reported no diurnal variation in intensity of bulbar weakness or fatiguability, a diagnosis of MG was not initially thought of. Instead, it was diagnosed as acute ischemic stroke that seemed like a probable partial Wallenberg (medullary) syndrome. A 1.5 tesla magnetic resonance imaging (MRI) of the brain with MR angiogram revealed no abnormalities. Considering a diffusion negative stroke, the patient was admitted; antiplatelets, low molecular weight heparin, neurotropics, antihypertensives and statins were commenced. Nasogastric (NG) tube insertion was planned to prevent aspiration of food. Routine blood investigations, thrombophilia blood workup, a 2D echocardiogram, 24-hour holter monitoring were ordered as part of young stroke workup, all of which yielded normal results. During hospital stay, the patient had difficulty in articulation of speech, elevated blood pressures on several occasions, persistent nasal speech and also developed mild breathlessness on the third day of admission. However, breathlessness gradually reduced after treating with nebulized bronchodilators and nebulized corticosteroids, his high blood pressure was controlled with optimization of antihypertensives.

Upon discharge after a week, the patient was doing better, but signs of bulbar weakness (hyponasal voice, nasal regurgitation) were persistent; thus, it was decided to continue feeding via nasogastric tube to prevent further aspiration of food. Upon review a week later, the patient initially reported having some improvements in his symptoms and this provided a false sense of assurance. During subsequent follow up consultation after a month, there was still noticeable palatal weakness and associated dysphagia. In view of persistent symptoms, a fluoroscopic evaluation of deglutition was conducted, which revealed weakness in the base of tongue and palatal muscles hindering normal deglutition. A flexible endoscopic study rendered the patient not fit for taking food orally. Thus, NG tube was kept in-situ. It is to be noted that antiplatelets and statins were being continued with good compliance.

**Figure 1:**
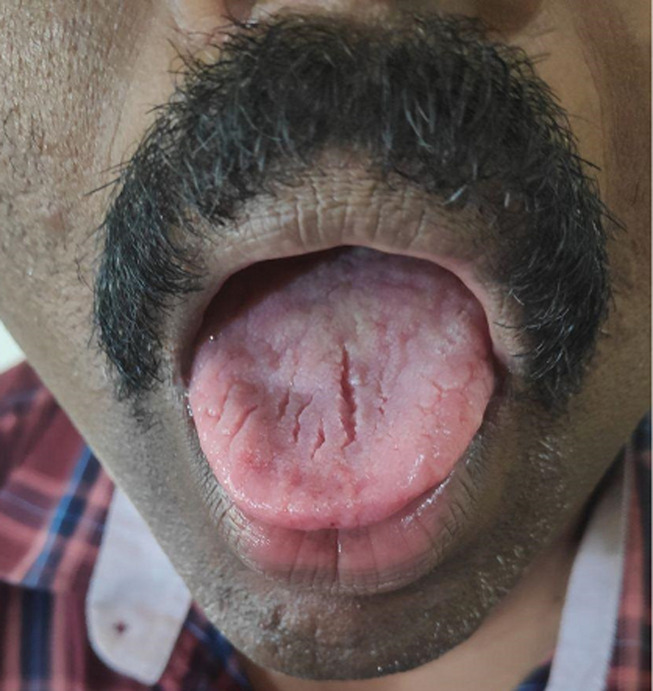
furrowed/fissured appearance of tongue with atrophy, found on examination of oral cavity before commencement of therapy

A month later, the patient presented with further worsening of symptoms, he was also noticed to have drooping of his eyelids, chewing difficulty with diurnal variation and increasing generalized fatiguability. Examination of oral cavity revealed a furrowed/fissured appearance of tongue with atrophy (Figure 1) as well. Keeping a probability of myasthenia gravis as a differential diagnosis, a repetitive nerve stimulation (RNS) test and estimation of acetyl choline receptor (AchR) antibody in serum were planned immediately. Along with that, a low dose of pyridostigmine too was commenced. As anticipated, the RNS was suggestive of generalised MG, AChR antibody was strongly positive (17nmol/L, normal-<0.4nmol/L). This was followed by the commencement of a full-fledged therapy with neostigmine (30mg), pyridostigmine (300mg) and oral prednisolone (10mg).

**Figure 2:**
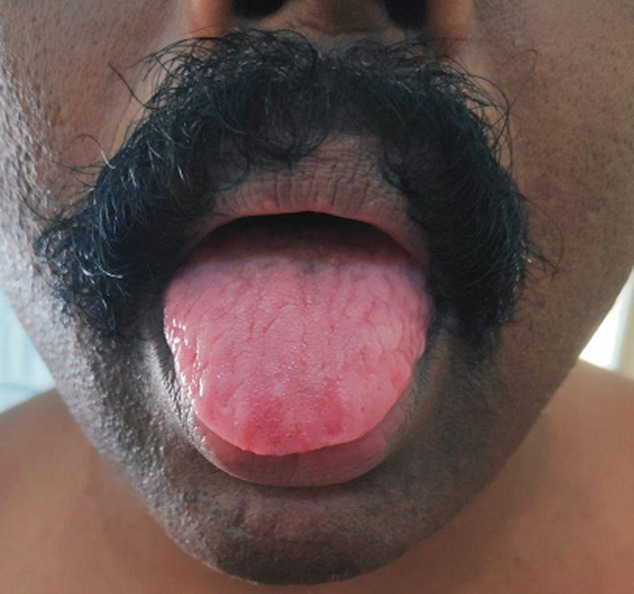
marked reduction in atrophy and furrowed/fissured appearance of tongue after therapy for 3 months

The patient was then reviewed in two weeks after performing a computerized tomography (CECT) scan of the thorax which fortunately revealed no thymoma. He showed significant improvement in his bulbar weakness and fatiguability. The same regimen was repeated for two more months resulting in marked reduction in symptoms, weaning him off his NG tube and he finally became symptom free by the end of three months. During review in the last week of March 2020, he was asymptomatic. On examination of his tongue, which was previously fissured/furrowed and atrophied (a rare finding in AchR antibody positive MG [1]), there was notable improvement (Figure 2) upon continuing the same line of management.

## Discussion

This patient´s clinical case reveals that MG can occasionally mimic the more common entity of stroke [2], these occurrences and the subsequent associated danger to patients in such cases have not been thoroughly addressed. Being a long-term neuromuscular disease, myasthenia gravis leads to varying degrees of skeletal muscle weakness. However, the most common complaints are ptosis, dysarthria, dysphagia, diplopia, weakness of extremities and respiratory weakness. The onset of symptoms can be sudden, with a relapsing and remitting course. Although it is a well-described entity with classic features, this disease may present with diagnostic difficulties in patients who have comorbid illnesses and vague symptoms, especially in elderly. Myasthenia gravis can cause significant morbidity, but it is a treatable neuromuscular disorder. There is disruption in neuromuscular transmission in which AChR antibodies cause autoimmune destruction of functioning AChRs on the postsynaptic membrane of the neuromuscular junction.

This case demonstrates how myasthenia gravis may present atypically without the classical fatigable weakness or diurnal variation, leading to misdiagnosis, prolonged or unwanted hospital admission, investigations and preventable health risks. Moreover, in a hyper acute scenario when stroke is suspected or misdiagnosed, neuroimaging may reveal a normal scan report. IV thrombolysis, if given, can lead to significant morbidity. In light of the high prevalence of silent cerebrovascular disease in elderly patients, neuro imaging findings may mislead clinicians who interpret incidental findings as diagnostic [2].

Finally, the triple furrow or fissured or trident tongue is a rare but characteristic manifestation of MG in which a midline and two parallel longitudinal grooves appear in the tongue, there is usually associated tongue atrophy. Muscle atrophy is atypical in MG except in the muscle specific tyrosine kinase (MuSK) variant, which is reversible with treatment [3].

## Conclusion

To sum up, when coming across cases of weakness, especially of cranial musculature, a meticulous neurological examination is mandatory. It is a well-known fact that brainstem infarcts can present with bulbar weakness resulting in depressed cough reflex and aspiration pneumonitis [4], as seen in the above case. Therefore, a differential diagnosis of MG should be kept in mind and confirmatory tests should be planned ahead. An early and correct diagnosis can avoid preventable complications from this treatable condition. Furthermore, there are numerous published documented reports of myasthenia gravis mimicking a stroke in elderly patients [2,5] but the same occurrence in younger age groups is quite rare; recent publications report an increased incidence of stroke in young adults [6]. The prevalence of standard modifiable vascular risk factors in young stroke patients is different from that in older patients. Modifiable risk factors for stroke, such as dyslipidemia, smoking and hypertension are highly prevalent in the young stroke population, with no significant difference in geographic, climatic, nutritional, lifestyle or genetic diversity [6]. Hence, due of these changing trends, chances of misdiagnosis even in younger population can potentially increase in the coming times.
